# Magnetic immunofluorescent microfluidic chips for rapid multi-parameter detection of serum antibodies to brucellosis and echinococcosis

**DOI:** 10.7717/peerj.21509

**Published:** 2026-07-03

**Authors:** Junhao Li, Yiran Wang

**Affiliations:** 1 School of Public Health, Xinjiang Second Medical College, Karamay, Xinjiang Uygur Autonomous Region, China; 2 School of Clinical Medicine, Xinjiang Second Medical College, Karamay, Xinjiang Uygur Autonomous Region, China

**Keywords:** Echinococcosis, Brucellosis, Microfluidic chip, Immunofluorescence

## Abstract

**Objectives:**

Brucellosis and echinococcosis are highly prevalent in pastoral areas, and traditional detection methods are limited because they are labor intensive, time consuming, and can only detect single-pathogens. This study aims to develop a magneto-immunofluorescent microfluidic chip for the rapid and simultaneous detection of serum antibodies against both brucellosis and echinococcosis and verify the detection performance and clinical application potential of the chip.

**Methods:**

In this study, a magneto-immunofluorescent microfluidic chip was designed, fabricated and optimized, with a focus on the conjugation ratios of *Echinococcus*/*Brucella* antigens/antibodies to microspheres and the binding ratios of samples to fluorescent microspheres. The 2D/3D structures of the chip were verified *via* computational fluid dynamics simulation, and the core detection performance of the chip (linear range, limit of detection (LOD), precision, and specificity) was validated using positive sera and reference materials. The detection consistency between the chip and the clinical enzyme-linked immunosorbent assay (ELISA) was evaluated by coincidence rate, Kappa test, and Bland-Altman analysis. The technical advantages and limitations of the chip were then summarized.

**Results:**

The chip stably and reliably detected eight target antibodies (AgB IgG, Ag5 IgG, Em2 IgG, Em18 IgG, LPS IgM, LPS IgG, OMP25 IgM, OPM25 IgG), with all key performance indicators meeting clinical requirements. It showed high consistency with ELISA: the positive and negative coincidence rates of all target antibodies exceeded 90%, Em18 IgG and LPS IgG achieved 100% positive coincidence rate, and Em18 IgG also had 100% negative coincidence rate; Kappa values ranged from 0.88 to 0.98 (far higher than the 0.75 threshold for good consistency), and more than 90% of samples fell within the 95% confidence interval in Bland-Altman analysis. Compared with ELISA, the chip had advantages of being a “one-chip multi-test”, with low sample consumption, rapid detection, and simple operation, suitable for on-site screening in pastoral areas.

**Conclusions:**

The magneto-immunofluorescent microfluidic chip developed in this study enables the rapid and simultaneous detection of antibodies against brucellosis and echinococcosis, featuring excellent detection performance and high consistency with the gold standard method. This chip is expected to provide critical technical support for the early diagnosis, epidemiological surveillance, and precise prevention and control of both brucellosis and echinococcosis, thereby improving public health and preventing economic losses in the animal husbandry industry in high-prevalence regions of these two diseases.

## Introduction

Brucellosis and echinococcosis are typical, globally prevalent zoonotic diseases that pose particularly severe threats to pastoral areas such as Xinjiang, Xizang, and Inner Mongolia in China (Qureshi et al., 2023; Jensenius et al., 2024; Shaheen, 2022). Brucellosis is induced by *Brucella* infection, and its acute stage is mainly characterized by sustained fever, migratory arthralgia, and reproductive system damage. Without timely and effective intervention, the disease easily progresses to a chronic state, triggering irreversible severe complications including chronic arthritis, endocarditis, and central nervous system impairment. Most chronic patients suffer from sequelae such as persistent fatigue, cognitive decline, and reduced quality of life, and patients with severe cases may die of multiple organ failure (Jin et al., 2023; Laine et al., 2023). Echinococcosis is caused by the parasitism of *Echinococcus* larvae, which mainly invade the liver and lungs, and can also affect vital organs, including the kidneys and brain. As the cysts grow, they compress surrounding tissues and organs and cause dysfunction; cyst rupture can induce fatal anaphylactic shock or secondary abdominal infection, and advanced patients often succumb to cachexia or severe complications (Lupia et al., 2021; Weber, Junghanss & Stojković, 2023).

Globally, brucellosis and echinococcosis impose a heavy burden on public health, livestock breeding, and socioeconomic development. More than 500,000 new cases of brucellosis occur worldwide each year, and echinococcosis is endemic in more than 70 countries across five continents (Khairullah et al., 2024; Ghasemirad et al., 2022). In livestock production, these two diseases reduce the fertility and survival rates of livestock and poultry, degrade the quality of livestock products, and cause substantial economic losses. In China alone, the annual economic losses to the animal husbandry industry caused by these two diseases reach tens of billions of yuan (Rostami et al., 2025; Tian et al., 2024). Furthermore, the long treatment cycle and high medical costs of these diseases significantly increase the burden on the healthcare system, which is a significant concern in resource-limited regions. These diseases have become major public health challenges restricting global public health security and the sustainable development of agriculture (Ghssein et al., 2025; Pavlidis, Galanis & Pavlidis, 2025).

In high-prevalence areas, co-infection with brucellosis and echinococcosis is common, creating an urgent demand for simultaneous screening technologies. Therefore, timely and accurate diagnosis is key to the prevention and control of these two diseases. Currently, commonly used clinical detection methods, such as enzyme-linked immunosorbent assay (ELISA), can detect antibodies, but they are labor intensive, slow (usually several hours), require a large consumption of samples and reagents, and do not meet the demand for on-site rapid detection (Hayrapetyan et al., 2023; Aydin et al., 2025). Meanwhile, single-pathogen detection methods are unable to screen for both diseases simultaneously, leading to low efficiency in epidemiological surveys and large-scale prevention and control efforts. Therefore, developing a rapid, sensitive, and specific dual antibody simultaneous detection technology has become a research focus in the field of zoonotic disease detection.

Microfluidic chip technology is characterized by miniaturization, integration, and automation, which can integrate the whole process of sample processing, reaction, and detection into micron-scale channels. It has the advantages of low sample consumption, fast detection speed, and strong portability, showing great application potential in the field of point-of-care testing (POCT) (Farhang Doost & Srivastava, 2024; Tu et al., 2022). For example, Wu et al. (2022) established a novel method for detecting carbapenemase-producing bacteria based on microfluidic chips, with a sensitivity of 97.7%; Kasputis et al. (2024) developed a microfluidic chip that can achieve rapid quantitative detection of Zika virus in specimens within one hour. Magnetic immunoisolation technology can efficiently enrich antibodies in samples and improve detection sensitivity by specifically capturing targets with magnetic microparticles (Schirra et al., 2023); immunofluorescence detection technology enables accurate quantification of trace targets (Zhong & Chen, 2023). The integration of magnetic immunofluorescence detection and microfluidic chip technology is expected to form a novel multiplex detection method for brucellosis and echinococcosis with both rapid screening and high-sensitivity detection capabilities.

Accordingly, this study intends to design and create a magnetic immunofluorescent microfluidic chip to achieve the rapid combined detection of serum antibodies against brucellosis and echinococcosis. By optimizing the chip structure and detection parameters, the linear range, limit of detection, precision, specificity, and accuracy of this method will be systematically verified, and a comparison will be made with the clinical gold standard ELISA method. This study is expected to provide novel technical support for the early diagnosis, epidemic monitoring, and precise prevention and control of these two zoonotic diseases.

## Materials & Methods

### Study samples

From January to July 2025, serum samples were collected in this study from pastoral areas in Ili Kazak Autonomous Prefecture, Tacheng Prefecture, Altay Prefecture, and the First Affiliated Hospital of Xinjiang Second Medical College, including 50 serum samples positive for *Echinococcus granulosus* AgB IgG, 50 samples positive for *Echinococcus granulosus* Ag5 IgG, 50 samples positive for *Echinococcus multilocularis* Em2 IgG, and 50 samples positive for *Echinococcus multilocularis* Em18 IgG. During the same period, positive serum samples related to *Brucella* were collected from the above-mentioned regions and medical institution, including 50 samples positive for *Brucella* lipopolysaccharide (LPS) IgM, 50 samples positive for *Brucella* LPS IgG, 50 samples positive for *Brucella* outer membrane protein 25 (Omp25) IgM, and 50 samples positive for *Brucella* Omp25 IgG.

Ethical approval: This study was conducted in strict accordance with the Declaration of Helsinki and approved by the Ethics Committee of Xinjiang Second Medical College (Approval No. MEC-XSMC-KT-20240630-001). A waiver of informed consent was granted by the above Ethics Committee for the conduct of this study.

Diagnostic basis: The clinical diagnosis of echinococcosis was performed in accordance with the Diagnostic Criteria for Echinococcosis (WS 257-2006) (Ministry of Health of the People’s Republic of China, 2006); the clinical diagnosis of brucellosis followed the Diagnosis of Brucellosis (WS 269-2019) (National Health Commission of the People’s Republic of China, 2019).

Inclusion criteria: (1) Positive results detected by enzyme-linked immunosorbent assay (ELISA) according to the corresponding diagnostic criteria mentioned above from (2) subjects aged over 18 years and under 80 years were included in the study.

Exclusion criteria: (1) Serum samples from echinococcosis patients complicated with other parasitic infections were excluded; (2) serum samples from brucellosis patients complicated with other common Gram-negative bacterial infections were excluded; (3) Serum samples from subjects who had received anti-parasitic treatment or antibacterial therapy in the past three months were excluded.

### Conjugation of antibodies and antigens to microspheres

Carboxyl-modified fluorescent microspheres (10 mg/mL, 500 nm; Zhongke Leiming) and carboxyl-modified magnetic beads (10 mg/mL, 500 nm; Thermo Fisher Scientific) were used as solid supports. Conjugation was performed *via* a standard EDC/NHS activation protocol. Briefly, one mL of carboxyl microspheres was activated by adding a total of 500 mg of 1-ethyl-3-(3-dimethylaminopropyl)carbodiimide (EDC) and N-hydroxysuccinimide (NHS) dissolved in 2-(N-morpholino)ethanesulfonic acid (MES) buffer at pH 6.0. After activation, fluorescent microspheres were incubated with anti-human IgG monoclonal antibody (five mg/mL; Merck) and anti-human IgM monoclonal antibody (five mg/mL; Merck) at 37 °C for 2 h to form covalent conjugates. Under the same incubation conditions, magnetic beads were coupled with a panel of target antigens at a concentration of 0.5 mg/mL each, including AgB, Ag5, Em2 and Em18 antigens (Yinduo Biotechnology), as well as lipopolysaccharide (LPS) and outer membrane protein 25 (Omp25) antigens (Hangzhou Yiminuo Biotechnology). Following conjugation, 3% (w/v) bovine serum albumin (BSA) solution was applied for 2 h at room temperature to block non-specific binding sites on the microspheres. The conjugated microspheres were then washed twice with phosphate-buffered saline (PBS) containing 0.05% Tween-20, resuspended in PBS supplemented with 0.1% BSA, and stored at 4 °C for subsequent experiments.

### Optimization of conjugation ratios

Conjugation ratios between microspheres and antibodies/antigens were optimized at five mass ratios: 10:1, 25:1, 50:1, 75:1, and 100:1. Each reaction system was mixed uniformly and incubated for 3 h with gentle stirring. After conjugation, the amount of free antibody/antigen in the supernatant was determined by ultraviolet spectrophotometry. The coupling efficiency was calculated as follows: coupling efficiency (%) = [(total initial antibody/antigen amount − free antibody/antigen amount in supernatant)/total initial antibody/antigen amount] ×100%. All tests were performed in triplicate, and the ratio showing the highest coupling efficiency was selected as the optimal parameter.

### Optimization of sample dilution and microsphere dosage

Serum samples were serially diluted with 0.01 M PBS (pH 7.4) containing 2% BSA and 0.05% Tween-20 at ratios of 1:50, 1:100, 1:200, and 1:400. Diluted samples were reacted with activated fluorescent microspheres at three dosage gradients: 1 µg, 5 µg, and 10 µg. After incubation, the fluorescence signal value and background value of each group were recorded. The signal-to-background ratio (S/B ratio) was calculated to evaluate the detection performance. The combination with the maximum S/B ratio was determined as the optimal condition. All experiments were repeated three times to ensure reliability.

### Structural design and chip fabrication

**Figure 1:**
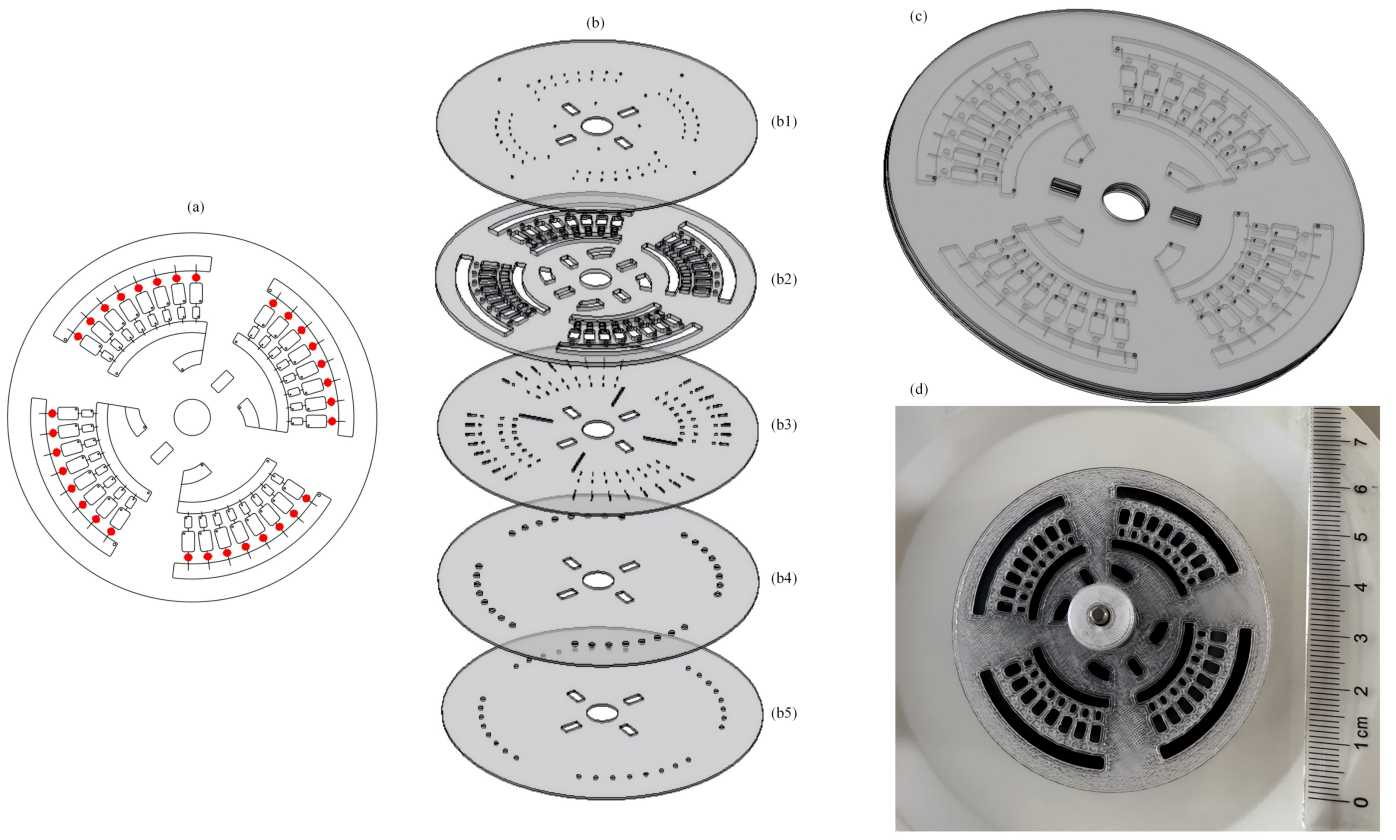
Schematic diagrams of the 2D and 3D structures of the chip. (A) Schematic diagram of the 2D structure of the chip; (B1) pressure stabilization layer of the chip; (B2) chamber reaction layer; (B3) microfluidic channel layer; (B4) valve control layer; (B5) base layer of the chip; (C) schematic diagram of the 3D structure of the chip; (D) physical image of the chip.

The two-dimensional (2D) and three-dimensional (3D) structures of the microfluidic chip were designed using professional design software, and the corresponding diagrams are displayed in Fig. 1. The chip adopts a five-layer integrated structure, which consists of (from top to bottom) the constant pressure layer, chamber reaction layer, microfluidic channel layer, valve control layer, and base layer. The functions of each layer are as follows: the constant pressure layer stabilizes the air pressure to ensure smooth liquid flow; the chamber reaction layer provides the reaction space for immunoreactions; the microfluidic channel layer enables the stable transmission of microfluidics; the valve control layer regulates the fluid flow into the waste pool; the base layer acts as the reaction substrate for antigen-antibody binding. The 2D schematic diagram of the chip is shown in Fig. 1A, the structural diagrams of each functional layer are presented in Figs. 1B1–1B5, and the overall 3D structure of the optimized chip is illustrated in Fig. 1C.

Microfluidic chips were fabricated by combining 3D printing and laser cutting processes in this study. The chip was designed with five functional layers, which were fabricated individually. The auxiliary structural components were manufactured using a Bambu Lab H2D 3D Printer (Bambu Lab Technology Co., Ltd., Shenzhen, China), and microchannels on the substrate were precisely etched with a TruMicro Series 5000 laser cutter (TRUMPF). After all functional layers were processed, a Sublym100™ microfluidic vacuum hot press (Eden-microfluidics) was used for thermal bonding and packaging. All layers were tightly laminated to form an integrated microfluidic chip with excellent sealing performance and structural stability. The physical appearance of the assembled chip is shown in Fig. 1D.

### Hydrodynamic simulation and validation

The chip adopts a disc-shaped microfluidic chip structure, and its symmetrical structure enables uniform liquid splitting. To verify whether this chip meets the fluid operation conditions, the fluid dynamics simulation software Comsol Multiphysics 6.5 was used to test the channel pressure, and the wall resolution was measured to ensure the authenticity and reliability of the simulation results.

Parameters for hydrodynamic tests were set, as follows: The serum density was set to 1,020 kg/m^3^ according to the research data of Craft, Epler & Butler (1993); the serum viscosity was set to 1.2 mPa s based on the research data of Rosenson, McCormick & Uretz (1996); the surface tension was set to 48 mN/m in accordance with the research data of Rosina, Kvasnák & Suta (2007); and the flow mode was set to laminar flow based on the research data of Duffy et al. (1999). The theoretical basis of the flow velocity calculation formula is derived from the Poiseuille flow principle combined with the radial pressure gradient generated by centrifugal force.

The core of centrifugal force-driven motion lies in the centrifugal acceleration generated by rotation, whose calculation formula is as follows:

(1) \begin{eqnarray*}\alpha ={\omega }^{2}r\end{eqnarray*}

where *ω* is the angular velocity (unit: rad/s). The rotational speeds adopted for this chip, which were determined based on our group’s experimental experience and preliminary tests, are 3.33 rad/s, 8.33 rad/s, 13.33 rad/s, and 16.67 rad/s; r is the rotational radius (unit: m), and the rotational radii corresponding to these four rotational speeds are 0.012 m, 0.016 m, 0.018 m, and 0.02 m, respectively.

All fluid channels of this chip have a rectangular cross-section, and the calculation formula for the liquid flow velocity in such channels is as follows:

(2) \begin{eqnarray*}\upsilon = \frac{\rho {h}^{2}\alpha }{12\mu } \end{eqnarray*}

where *ρ* is the fluid density (unit: kg/m^3^), which is known; h is the channel height (unit: m), with a value of 0.0008 m; *α* is the centrifugal acceleration (unit: m/s^2^), calculated by Eq. (1); and *μ* is the dynamic viscosity of the fluid (unit: Pa s), which is known.

After calculating the liquid flow velocity in the chip using the above formula, hydrodynamic simulation tests were conducted using the above parameters.

### Operational procedure and judgment criteria

Enzyme-linked immunosorbent assay (ELISA) was used as the reference method. For each of the eight detection targets (AgB IgG, Ag5 IgG, Em2 IgG, Em18 IgG, LPS IgM, LPS IgG, Omp25 IgM, Omp25 IgG), 50 positive and 50 negative commercial standard serum samples were tested. The optimal cut-off value for the microfluidic chip was determined by receiver operating characteristic (ROC) curve analysis. Samples with detection values higher than the cut-off value were judged as positive, and those below the threshold were judged as negative.

**Figure 2:**
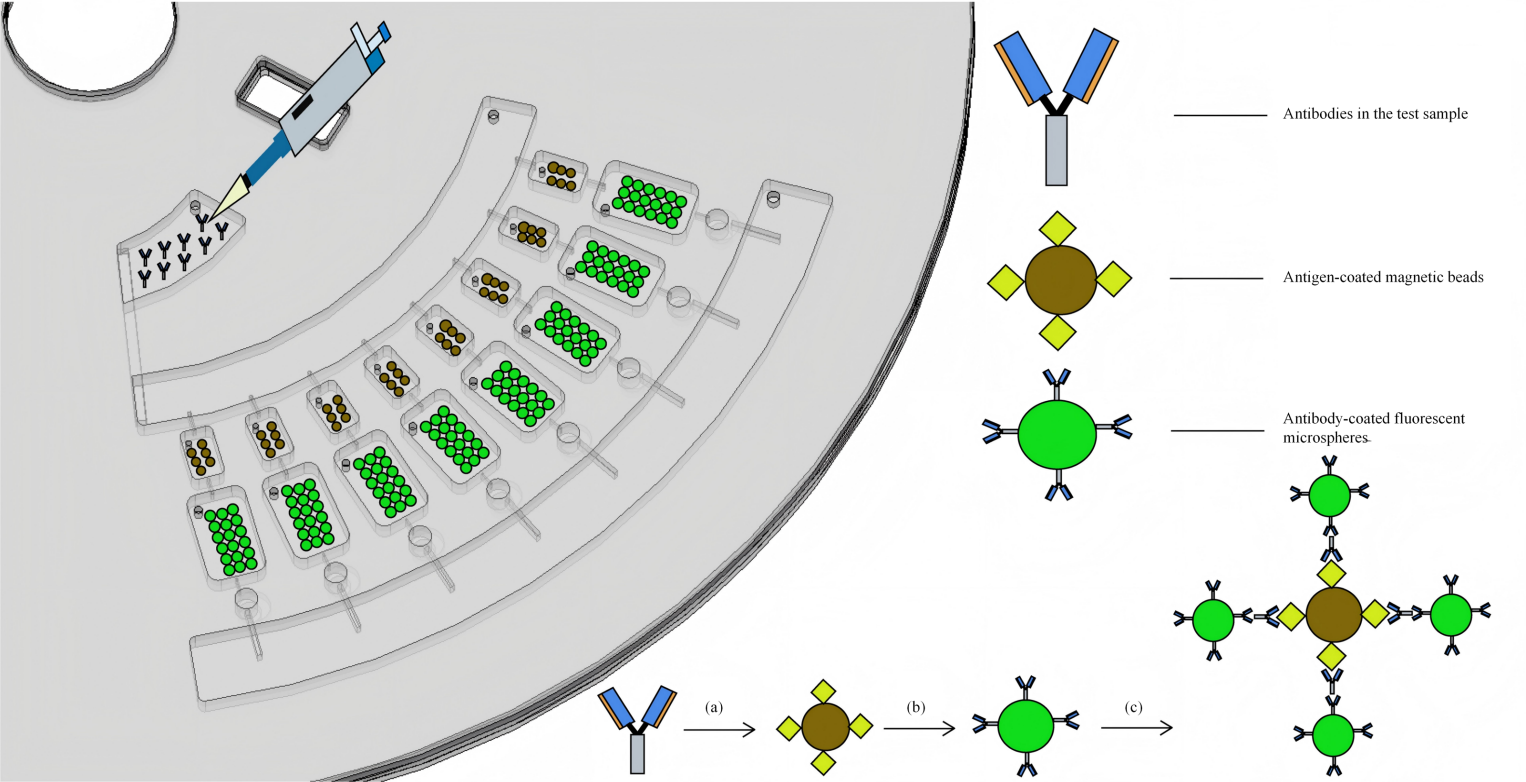
Working process diagram of the microfluidic chip. (A) First incubation and washing step; (B) second incubation and washing step; (C) detection step.

After sample loading, the serum sample was transferred into the antigen-magnetic bead storage chamber assisted by centrifugal force and the distribution reservoir. After incubation and washing, antibodies in the sample bound to *Echinococcus* or *Brucell* a antigens on magnetic beads to form magnetic bead-antigen-antibody immune complexes. Subsequently, the complexes were driven into the detection chamber by centrifugation. After another round of incubation and washing, the complexes bound to secondary antibody-conjugated fluorescent microspheres to form magnetic bead-antigen-antibody-fluorescent microsphere sandwich complexes. Excess waste liquid was collected into the waste reservoir *via* centrifugation. Fluorescence detection was performed in the detection chamber. The fluorescence signals were collected, processed, and analyzed to obtain the final antibody detection results of serum samples. The detailed operational procedure is illustrated in Fig. 2.

### Methodological validation

#### Comparative analysis with ELISA

A total of 800 serum samples were used for comparative detection, including 50 positive and 50 negative samples for each of the eight targets (AgB IgG, Ag5 IgG, Em2 IgG, Em18 IgG, LPS IgM, LPS IgG, Omp25 IgM, Omp25 IgG). All samples were detected in parallel by the established microfluidic chip method and conventional enzyme-linked immunosorbent assay (ELISA). Each test was performed in triplicate to reduce experimental errors. Statistical analysis was conducted to verify the reliability of the chip-based detection results.

#### Dose–response curve and standard curve establishment

The standard samples derived from the echinococcosis and brucellosis ELISA kits (Beijing Tianzhitai Biotechnology) were used for serial dilution. The stock standard solution at an initial concentration of 100 µg/mL was gradiently diluted with the dedicated kit diluent to prepare six calibrated concentration levels: 0 (pure diluent as blank control), 6, 12, 24, 48, and 96 µg/mL. All standard samples were measured by the microfluidic chip, with three parallel replicates arranged for each concentration, and the corresponding fluorescence intensity values were recorded. Subsequently, a dose–response standard curve was established by plotting fluorescence intensity as the ordinate and standard concentration as the abscissa.

#### Limit of detection (LOD)

The 0 µg/mL standard was tested in 10 parallel replicates, and the fluorescence intensity was measured to calculate the mean value and standard deviation (SD). The LOD of this method was defined as the concentration calculated by substituting (mean + 2SD) into the standard curve equation.

#### Cut-off value determination

Based on the detection results of healthy human negative reference serum, the positive cut-off value was determined using the mean + 2SD method to ensure the scientificity and rationality of the result judgment.

#### Accuracy

Three control groups were set: positive control, negative control, and blank control. Positive and negative controls were derived from the matched standards of commercial ELISA kits, and the blank control was prepared with phosphate-buffered saline (PBS). All controls were detected separately, and the results were observed under a fluorescence microscope in bright field and dark field. Each control was tested in triplicate to verify the detection accuracy of the chip.

#### Precision

High-concentration and low-concentration samples confirmed by clinical ELISA were selected and repeatedly detected 10 times by the microfluidic chip, respectively. Precision parameters were calculated based on the mean value and relative standard deviation (RSD) of the 10 repeated results.

#### Specificity

The specificity of the chip was evaluated by cross-detection using commercial standard sera, including five replicates of positive sera for each target (AgB IgG, Ag5 IgG, Em2 IgG, Em18 IgG, LPS IgM, LPS IgG, Omp25 IgM, Omp25 IgG), healthy human negative serum, and positive sera for cross-reactivity detection (*Toxoplasma gondii* IgG, *Cryptosporidium* IgG, *Escherichia coli* LPS IgG, *Salmonella* O-antigen IgG). Specificity indicators were calculated according to the presence or absence of cross-reactivity.

### Statistical analysis

Data sorting and preprocessing were performed using Microsoft Excel. All statistical analyses were carried out using GraphPad Prism 10 software. The Kappa test was used to evaluate the qualitative concordance of detection results between the microfluidic chip and ELISA methods. Linear regression analysis and Bland–Altman analysis were performed to assess the quantitative consistency. The consistency evaluation criteria were defined as follows: coefficient of determination (R^2^) ≥ 0.95 for linear regression; proportion of samples within the 95% confidence interval ≥ 85% for Bland–Altman analysis; Kappa value >0.8. Meeting all the above criteria indicated excellent consistency between the two methods and statistically reliable results.

## Results

### Design of optimization schemes for the reaction system

To enhance the sensitivity and specificity of the detection method developed in this study, systematic optimization was performed on the key parameters of the reaction system. The optimized indices included the conjugation ratio of anti-human IgG antibodies to carboxyl fluorescent microspheres, the conjugation ratio of antigens to carboxyl magnetic beads, and the binding conditions of samples to carboxyl fluorescent microspheres conjugated with anti-human IgG antibodies. The gradient optimization protocols and complete experimental data for all parameters are provided in Tables 1 and 2.

For the optimization of the conjugation efficiency of anti-human IgG antibodies to carboxyl fluorescent microspheres, five weight ratio gradients (10:1, 25:1, 50:1, 75:1, 100:1) were set up to conduct parallel comparative experiments. The results showed that at a weight ratio of 25:1, the content of free antibodies in the supernatant was 1.06 µg, with the conjugation efficiency reaching 89.4%—the highest value across all gradients. When the weight ratio exceeded 25:1 and increased continuously, the conjugation efficiency exhibited a gradual decreasing trend. For the conjugation system of antigens to carboxyl magnetic beads, the same five weight ratio gradients were adopted for optimization, and the results indicated a distinct variation pattern of conjugation efficiency compared with the former system. At a weight ratio of 50:1, the content of free antigens in the supernatant was only 1.09 µg, and the conjugation efficiency hit 89.1%, which was significantly higher than that of the other gradient groups. This ratio was thus identified as the optimal conjugation ratio for the antigen-carboxyl magnetic bead system.

On the basis of the optimized conjugation ratios, further optimization and screening of the sample dilution ratio and fluorescent microsphere dosage were carried out. Using the specific fluorescence signal intensity and the ratio of fluorescence intensity to background fluorescence as the core evaluation indices, a cross-combination experiment was designed with four gradient levels of sample dilution ratios (1:50, 1:100, 1:200, 1:400) and three gradient levels of fluorescent microsphere dosages (1 µg, 5 µg, 10 µg). The results demonstrated that different combinations of dilution ratios and microsphere dosages exerted significant differences in their effects on the detection signal. Specifically, at a sample dilution ratio of 1:200 and a fluorescent microsphere dosage of five µg, the specific fluorescence intensity of the detection system was 11.67 with a background fluorescence value of only 1.19, leading to a ratio of fluorescence intensity to background fluorescence of 9.8—the peak value among all experimental combinations. Under this condition, the detection system yielded the most specific detection signal with the lowest level of background interference.

**Table 1:** Optimization results of binding ratio between anti-human IgG antibody-modified carboxyl fluorescent microspheres and antigen-modified carboxyl magnetic beads.

**Range of weight ratios**	**Total initial amount of antibody (*μ*g)**	**Amount of free antibody in the supernatant (*μ*g)**	**Conjugation efficiency (anti-human IgG antibody conjugated to carboxyl fluorescent microspheres) (%)**	**Total initial amount of antigen (*μ*g)**	**Tmount of free antigen in the supernatant (*μ*g)**	**Conjugation efficiency (antigen conjugated to carboxyl magnetic beads) (%)**
10:1	10	2.79	72.1	10	3.41	65.9
25:1	10	1.06	89.4	10	2.78	72.2
50:1	10	1.23	87.7	10	1.09	89.1
75:1	10	2.08	79.2	10	2.77	72.3
100:1	10	2.49	75.1	10	2.54	74.6

**Table 2:** Optimization results of binding ratio between test samples and anti-human IgG antibody-coupled carboxyl fluorescent microspheres.

**Range of dilution ratios**	**Dosage of microspheres**	**Fluorescence intensity**	**background fluorescence**	**Fluorescence intensity/ background fluorescence**
1:50	1 ug	7.94	1.56	5.09
	5 ug	17.89	2.98	6
	10 ug	18.84	3.46	5.45
1:100	1 ug	6.57	1.09	6.03
	5 ug	14.89	2.27	6.56
	10 ug	16.03	3.06	5.12
1:200	1 ug	5.89	1.09	5.24
	5 ug	11.67	1.19	9.8
	10 ug	12.93	1.56	8.29
1:400	1 ug	5.43	1.03	5.27
	5 ug	6.89	1.22	5.65
	10 ug	8.03	1.94	4.14

Synthesizing the results of all the optimization experiments, the optimal reaction conditions of the detection system were determined as follows: a conjugation weight ratio of 25:1 for anti-human IgG antibodies to carboxyl fluorescent microspheres, a conjugation weight ratio of 50:1 for antigens to carboxyl magnetic beads, a sample dilution ratio of 1:200, and a fluorescent microsphere dosage of five µg.

### Structural design and practical application support of the microfluidic chip

In this study, a five-layer integrated microfluidic chip was independently designed and fabricated. As the core hardware of the entire detection system, this chip uses an integrated design to meet the core requirements of target detection and provides critical support for subsequent immunoreactions and fluorescence detection.

Through the rational design of functional partitions, the microfluidic chip constitutes a key foundation for the whole detection system. Its integrated five-layer structure realizes the integrated functions of air pressure stabilization, reaction loading, fluid transmission, valve control regulation, and substrate support, which enables the entire process of immunoreaction to be completed without additional auxiliary devices. In addition, the design of each functional layer is precisely matched with the reagent dosage and operational procedures of subsequent experiments, thereby ensuring the smooth progress of immunoreactions and the stability of the detection process.

The successful implementation of subsequent experiments and target detection is realized based on the designed functions of the chip, which further demonstrates the rationality and practicability of the chip design. This chip is the key hardware foundation for the stable performance of experiments in the detection system established in this study.

### Hydrodynamic simulation results and physical object display

**Figure 3:**
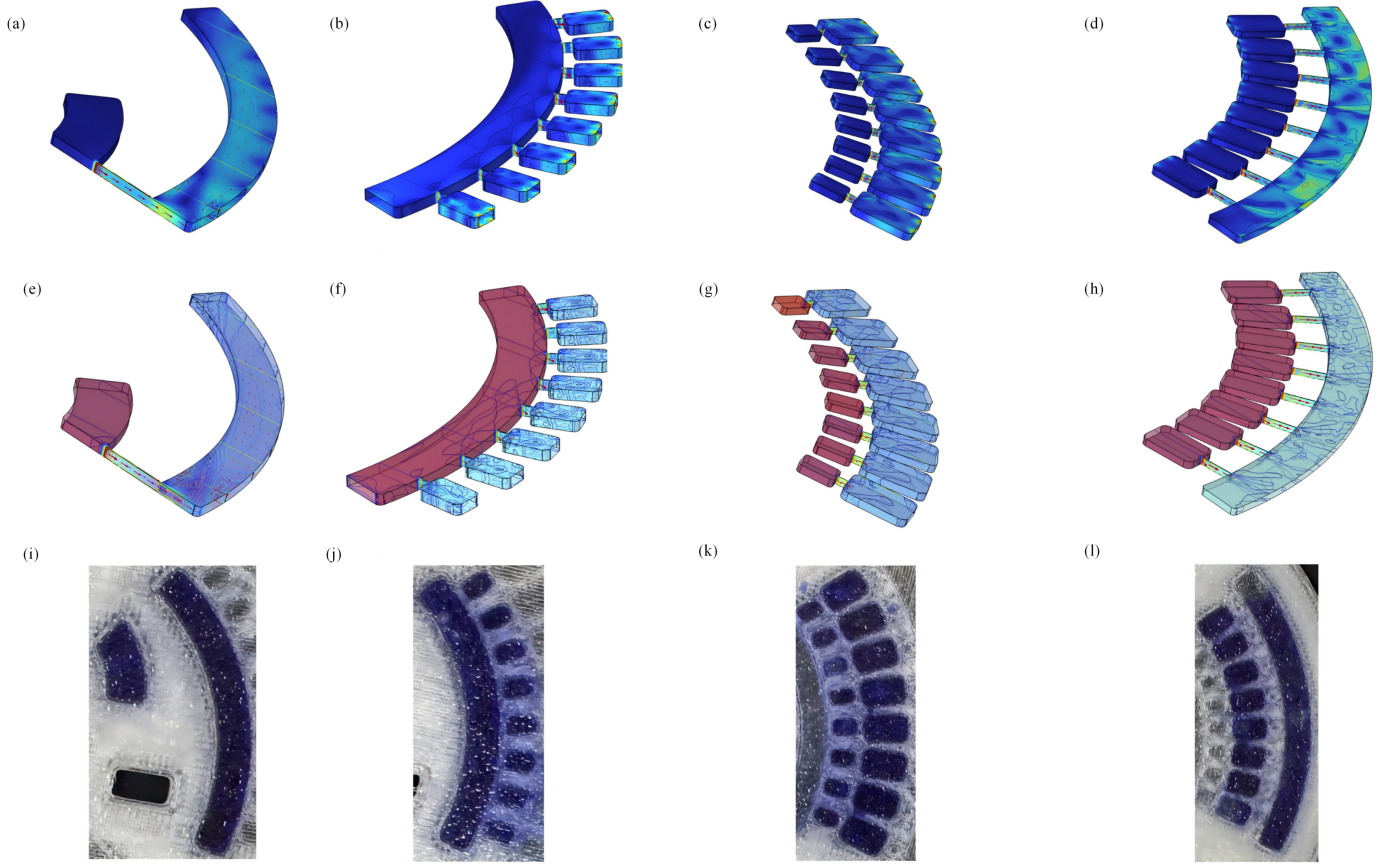
Computational fluid dynamics (CFD) simulation and physical fluid test results of the magneto-immunofluorescent microfluidic chip. Fluid flow simulations were performed at constant flow rates of 231 µL/min (sample loading chamber to distribution chamber), 1,933 μL/min (distribution chamber to magnetic bead chamber), 5,568 μL/min (magnetic bead chamber to detection chamber), and 9,675 μL/min (detection chamber to waste chamber). Physical verification was conducted using methylene blue dye under identical experimental conditions. (A)–(D) CFD simulation results of fluid pressure in the connecting channels between the sample loading chamber, distribution chamber, magnetic bead chamber, detection chamber, and waste chamber, respectively, demonstrating stable fluid transmission. (E)–(H) CFD simulation results of wall resolution for the aforementioned connecting channels, all of which meet the experimental requirements. (I)–(L) Physical verification images of fluid flow tests for the above connecting channels, respectively, indicating stable fluid operation.

Figure 3 presents hydrodynamic simulation results and physical photographs of the microfluidic chip. Computational fluid dynamics (CFD) analyses of four key fluid operation stages were carried out using COMSOL Multiphysics 6.5 to investigate the pressure distribution and wall shear stress characteristics within each structure of the chip. Pressure and wall shear stress remained stable throughout the fluid operation process, with no abnormal fluctuations or attenuation, satisfying the design requirements. Notably, the simulated pressure distribution and wall shear stress profiles were consistent with the flow patterns experimentally measured on the physical chip, showing no obvious discrepancies. Meanwhile, the fluid coverage area predicted by simulation closely matched the experimentally observed fluid region of the physical chip, and complete liquid filling was realized in all chambers. These results confirm that the CFD model can accurately characterize the flow performance of the chip, verify the scientific rationality of the structural design, and offer technical support for subsequent antibody detection experiments.

### Testing of chip detection time and accuracy

#### Chip detection time

The average time for sample loading is 1 min, with the first reaction averaging 5 min and the first washing step averaging 2 min. The second reaction lasts 5 min, followed by a second washing step averaging 2 min, and the final detection step takes 1 min on average. In total, the entire detection process is completed within 16 min.

**Figure 4:**
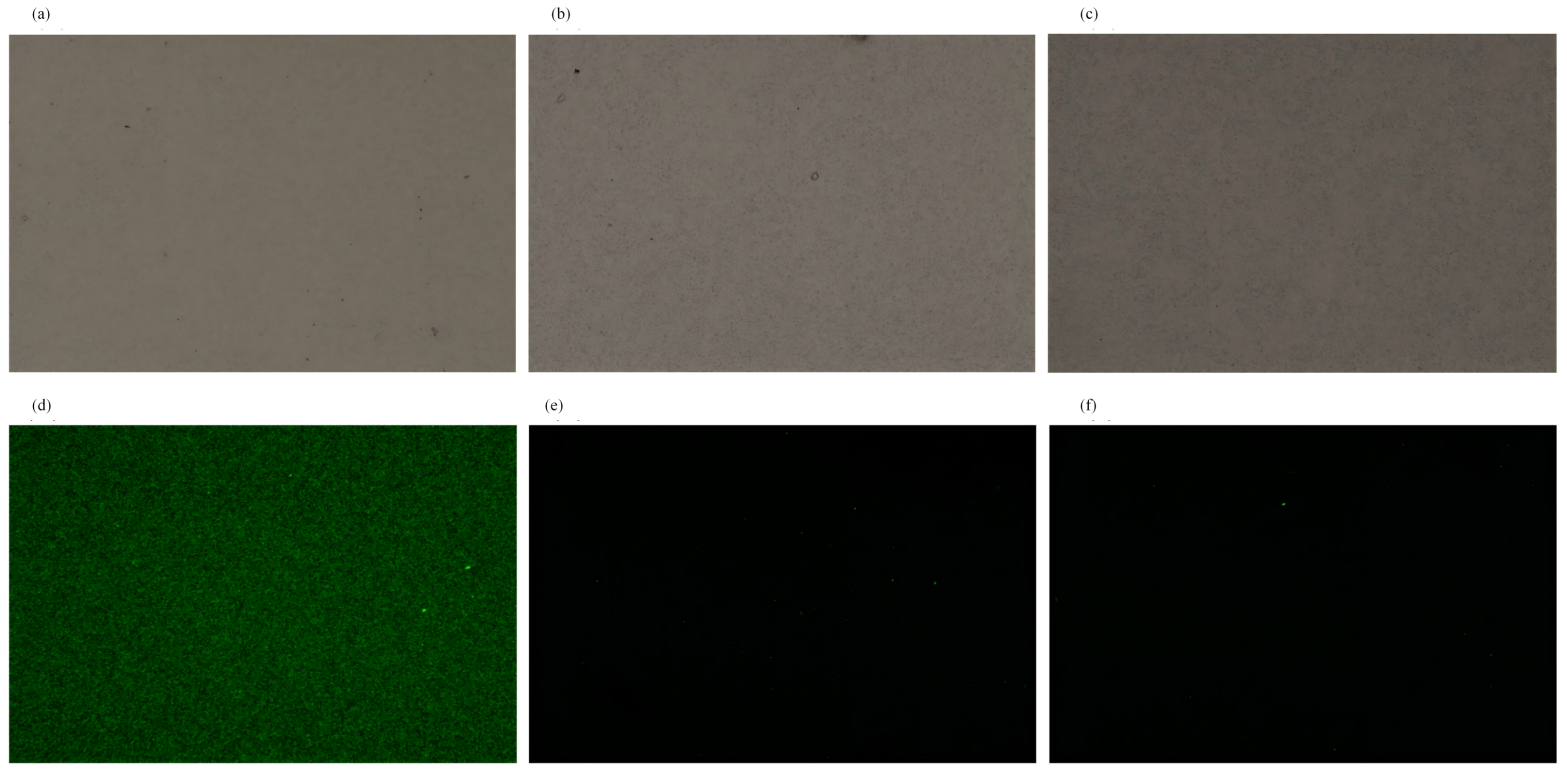
Representative bright-field and dark-field fluorescence microscopy images for the performance verification of the detection system. (A) Bright-field image of the positive control (with magnetic bead adsorption); (B) bright-field image of the blank control (with magnetic bead adsorption); (C) bright-field image of the negative control (with magnetic bead adsorption); (D) dark-field fluorescence image of the positive control (showing uniform full-field specific fluorescence); (E) dark-field fluorescence image of the blank control (no visible fluorescence); (F) dark-field fluorescence image of the negative control (no visible fluorescence).

In this study, the performance of the established detection system was verified using three control groups, and the corresponding results of bright-field and dark-field fluorescence microscopy are shown in Fig. 4. As confirmed by three repeated experiments, uniform and intense full-field fluorescence signals were stably observed in the positive control group under dark field, whereas no obvious fluorescence signals were detected in the dark fields of either the blank control group or the negative control group. In the bright-field images, significant magnetic bead enrichment and adsorption were observed in all three control groups. These consistent experimental results confirm that the detection chip passed performance verification and can be stably applied to subsequent experiments and practical detection.

### Optimal cutoff value test

**Figure 5:**
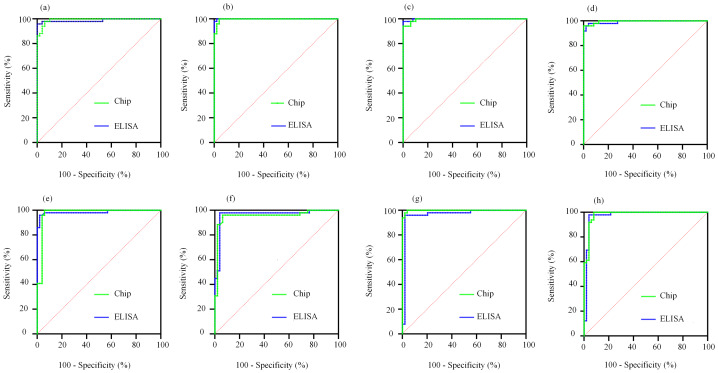
ROC curve analysis graphs. (A) ROC analysis graph of AgB IgG; (B) ROC analysis graph of Ag5 IgG; (C) ROC analysis graph of Em2 IgG; (D) ROC analysis graph of Em18 IgG; (E) ROC analysis graph of LPS IgM; (F) ROC analysis graph of LPS IgG; (G) ROC analysis graph of Omp25 IgM; (H) ROC analysis graph of Omp25 IgG.

Receiver operating characteristic (ROC) curve analysis was applied to determine the optimal cut-off values of the eight detection indicators for the microfluidic chip (AgB IgG, Ag5 IgG, Em2 IgG, Em18 IgG, LPS IgM, LPS IgG, Omp25 IgM, and Omp25 IgG). The ROC curves for each indicator are presented in Fig. 5, and the detailed ROC analysis results are summarized in Table 3.

The results showed that the optimal cut-off values of the eight indicators were concentrated in a narrow range of 5.2–6.5 µg/mL, presenting favorable consistency in these threshold levels. In terms of detection performance, all indicators achieved high sensitivity and specificity: the sensitivity ranged from 94% to 100%, and the specificity was stably maintained at 92%–100%. The area under the ROC curve (AUC) of each indicator was higher than 0.9557, with Omp25 IgM exhibiting the highest AUC value of 0.9984.

These results fully indicated that all detection indicators of the chip had an excellent ability to differentiate positive samples from negative ones. In conclusion, the optimal cut-off values determined by ROC curve analysis in this study are scientifically valid and reliable, so they can provide an accurate and effective judgment basis for the qualitative detection of the microfluidic chip.

**Table 3:** Cut-off value, sensitivity, specificity and AUC of different antibody indicators for diagnosis.

**Item**	**Cutoff (*μ*g/mL)**	**Sensitivity (%)**	**Specificity (%)**	**AUC**
AgB IgG	5.20	98.00	94.00	0.9928
Ag5 IgG	6.00	100.00	96.00	0.9968
Em2 IgG	6.50	94.00	100.00	0.9956
Em18 IgG	6.40	96.00	100.00	0.9960
LPS IgM	6.26	96.00	96.00	0.9760
LPS IgG	6.14	96.00	94.00	0.9557
Omp25 IgM	6.13	98.00	98.00	0.9984
Omp25 IgG	5.80	100.00	92.00	0.9816

### Dose–response curve and limit of detection test

**Figure 6:**
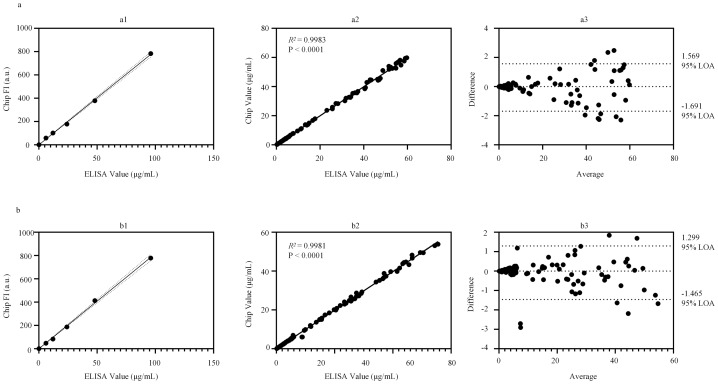
Dose–response curves, linear regression analysis plots, and Bland–Altman consistency analysis plots for the AgB IgG and Ag5 IgG assays. (A) Results plot for the AgB IgG assay: (A1) dose-response curve, (Sample Size, *N* = 3) (A2) linear regression analysis (Sample Size, *N* = 100; Correlation Coefficient, *R*^2^ = 0.9983), and (A3) Bland–Altman consistency analysis for the AgB IgG assay. (B) Results plot for the Ag5 IgG assay (Sample Size, *N* = 100; Mean bias = −0.0607): (B1) dose-response curve (Sample Size, *N* = 3), (B2) linear regression analysis (Sample Size, *N* = 100; Correlation Coefficient, *R*^2^ = 0.9981), and (B3) Bland–Altman consistency analysis for the Ag5 IgG assay (Sample Size, *N* = 100; Mean bias = −0.0829).

**Figure 7:**
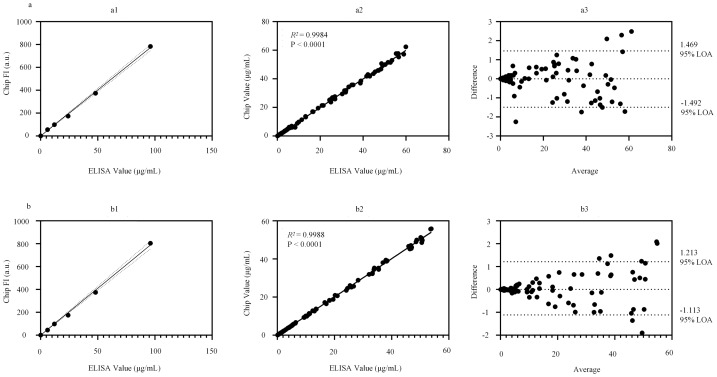
Dose–response curves, linear regression analysis plots, and Bland–Altman consistency analysis plots for the Em2 IgG and Em18 IgG assays. (A) Results plot for the Em2 IgG assay: (A1) dose-response curve (Sample Size, *N* = 3), (A2) linear regression analysis (Sample Size, *N* = 100; Correlation Coefficient, *R*^2^ = 0.9984), and (A3) Bland–Altman consistency analysis for the Em2 IgG assay (Sample Size, *N* = 100; Mean bias = −0.0116). (B) Results plot for the Em18 IgG assay: (B1) dose-response curve (Sample Size, *N* = 3), (B2) linear regression analysis (Sample Size, *N* = 100; Correlation Coefficient, *R*^2^ = 0.9988), and (B3) Bland–Altman consistency analysis for the Em18 IgG assay (Sample Size, *N* = 100; Mean bias = 0.0499).

**Figure 8:**
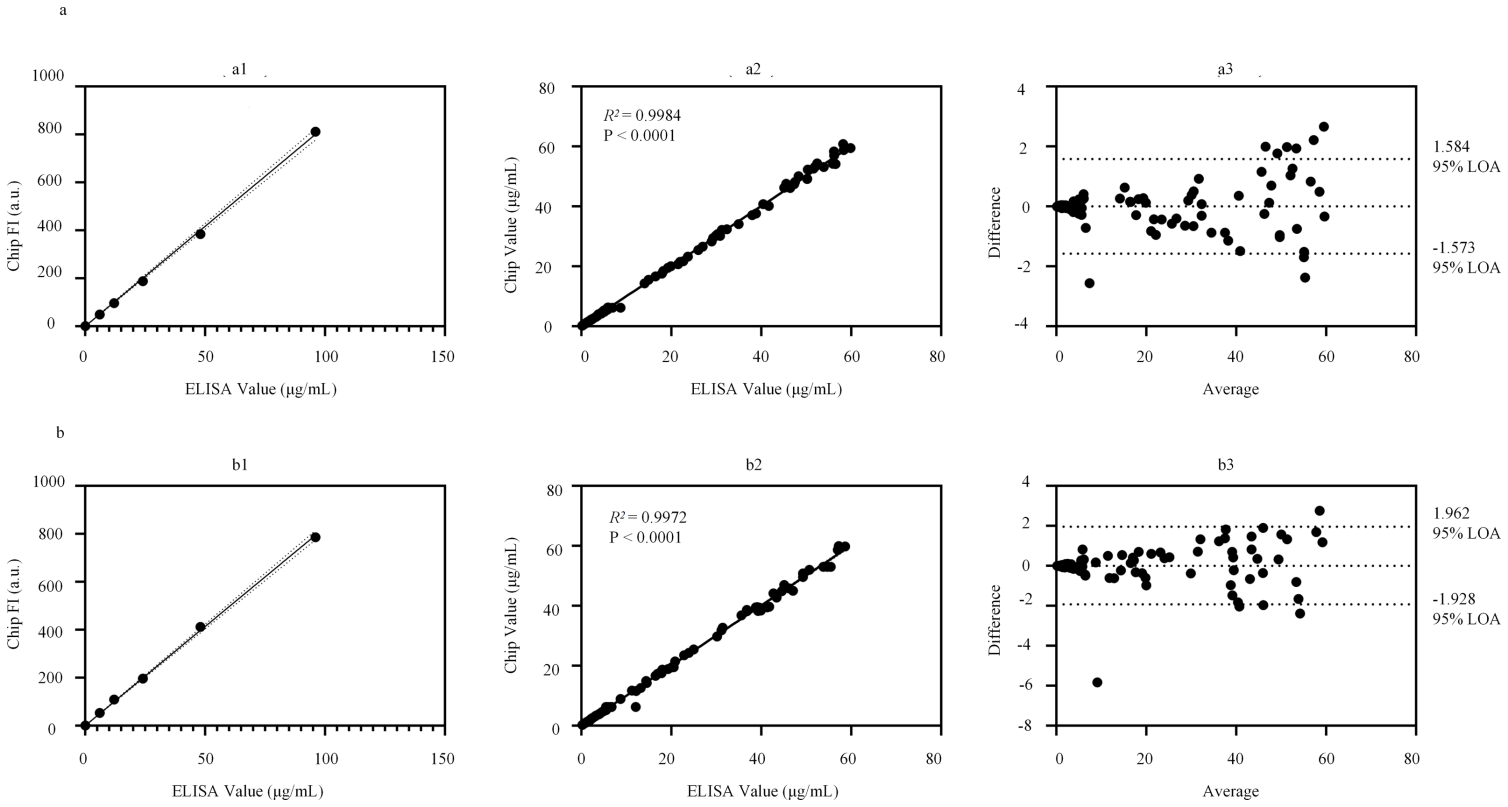
Dose–response curves, linear regression analysis plots, and Bland–Altman consistency analysis plots for the LPS IgM and LPS IgG assays. (A) Results plot for the LPS IgM assay: (A1) dose-response curve (Sample Size, *N* = 3), (A2) linear regression analysis (Sample Size, *N* = 100; Correlation Coefficient, *R*^2^ = 0.9984), and (A3) Bland–Altman consistency analysis for the LPS IgM assay. (B) Results plot for the LPS IgG assay (Sample Size, *N* = 100; Mean bias = 0.0047): (B1) dose-response curve, (B2) linear regression analysis (Sample Size, *N* = 100; Correlation Coefficient, *R*^2^ = 0.9972), and (B3) Bland–Altman consistency analysis for the LPS IgG assay (Sample Size, *N* = 100; Mean bias = 0.0171).

**Figure 9:**
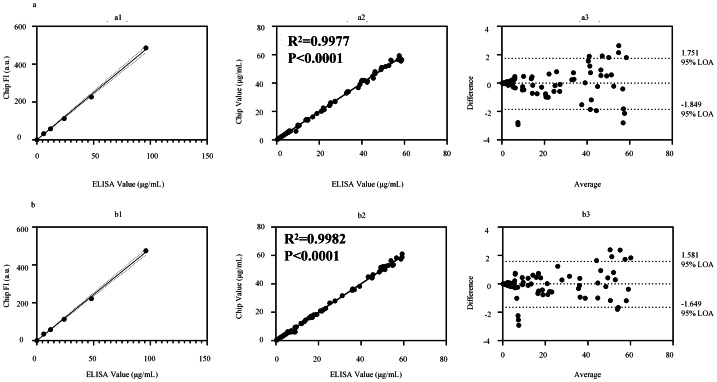
Dose–response curves, linear regression analysis plots, and Bland–Altman consistency analysis plots for the Omp25 IgM and Omp25 IgG assays. (A) Results plot for the Omp25 IgM assay: (A1) dose-response curve (Sample Size, *N* = 3), (A2) linear regression analysis (Sample Size, *N* = 100; Correlation Coefficient, *R*^2^ = 0.9977), and (A3) Bland–Altman consistency analysis for the Omp25 IgM assay (Sample Size, *N* = 100; Mean bias = −0.0488). (B) Results plot for the Omp25 IgG assay: (B1) dose-response curve (Sample Size, *N* = 3), (B2) linear regression analysis (Sample Size, *N* = 100; Correlation Coefficient, *R*^2^ = 0.9982), and (B3) Bland–Altman consistency analysis for the Omp25 IgG assay (Sample Size, *N* = 100; Mean bias = −0.0339).

In this study, quantitative detection was conducted for eight detection indicators of the microfluidic chip, namely AgB IgG, Ag5 IgG, Em2 IgG, Em18 IgG, LPS IgM, LPS IgG, Omp25 IgM, and Omp25 IgG. The dose–response curves of each indicator were fitted, and their linear regression equations, limits of detection (LOD), and linear detection ranges were determined accordingly. The detailed fitting equations, coefficients of determination (R^2^), linear ranges, and limits of detection (LOD) are presented in Table 4 and Figs. 6–9.

The results showed that all eight indicators exhibited favorable linear correlation within the preset concentration ranges, and the R^2^ values of their linear regression equations were all higher than 0.981. Among the indicators, LPS IgG achieved the optimal R^2^ value of 0.9993, which fully demonstrated that the dose–response curves of all indicators had a high degree of linear fitting. All indicators shared a unified upper limit of linear detection at 96 µg/mL, and their lower limits of linear detection ranged from 0.08 to 0.14 µg/mL.

These results confirmed that the microfluidic chip possessed a wide linear detection range, favorable linear correlation, and high detection sensitivity in quantitative detection. The characteristics of the dose–response curves and the overall detection performance of the chip all met the preset design specifications, providing reliable technical support for the subsequent application of the chip in the quantitative detection of actual samples.

### Repeatability test

A repeatability test was carried out for the eight detection indicators of the microfluidic chip. Two samples with low and high concentrations were selected for multiple parallel determinations, and the relative standard deviation (RSD) was used as the evaluation index to analyze the detection repeatability of the chip. The detailed mean detection values and RSD values of each indicator in different samples are presented in Table 5. The results indicated that the detection RSD values of all eight indicators at the two concentration gradients ranged from 2.78% to 5.77%. Among the indicators, Em18 IgG exhibited the best repeatability in the low-concentration sample with an RSD value of only 2.78%; Omp25 IgM had the highest RSD value among all indicators in the high-concentration sample, which was merely 5.77%. No obvious dispersion was observed in the determination results of all detection indicators. These results fully verified that the microfluidic chip possessed favorable repeatability and detection stability for the detection of samples with different concentrations, and its overall detection precision met the preset design criteria, indicating the chip would provide stable and reliable test results in subsequent sample detection work.

**Table 4:** Standard curve equation, determination coefficient, linear range and limit of detection of various detection indicators.

**Item**	**Dose–response curve equation**	*R* ^2^	**Linear range** **(*μ*g/mL)**	**Limit of detection (LOD,** ***μ*g/mL)**
AgB IgG	y = 8.1082x − 1.3337	0.9988	0.12–96.00	0.12
Ag5 IgG	y = 8.2321x − 3.6734	0.9983	0.13–96.00	0.13
Em2 IgG	y = 8.1119x − 4.0203	0.9984	0.09–96.00	0.09
Em18 IgG	y = 8.3662x − 9.9617	0.9981	0.14–96.00	0.14
LPS IgM	y = 8.4445x − 6.8320	0.9991	0.08–96.00	0.08
LPS IgG	y = 8.1954x + 5.3837	0.9993	0.13–96.00	0.13
Omp25 IgM	y = 5.0330x − 3.4606	0.9985	0.08–96.00	0.08
Omp25 IgG	y = 4.9021x − 1.6763	0.9984	0.13–96.00	0.13

### Specificity test

A specificity verification assay was performed for the eight detection indicators of the microfluidic chip. The results demonstrated that the chip successfully achieved positive detection for the specific samples corresponding to each of the eight indicators. No cross-reactivity was observed for all non-target indicators, and consistent negative detection results were obtained for these non-target indicators. A precise one-to-one positive matching relationship was established between each target indicator and its corresponding specific sample, and no non-specific binding or cross-detection events were observed throughout the entire assay process. These findings fully verified that the microfluidic chip possessed excellent detection specificity, which enabled the accurate and specific identification and detection of each target indicator without cross-reactivity. The specific detection performance of the chip fully met the preset design specifications, thereby providing a solid technical guarantee for the subsequent specific detection of actual samples.

### Analysis of consistency with existing detection methods

In this study, Bland-Altman analysis, Kappa test, and linear regression analysis were used together to evaluate the consistency of detection results of all antibody indicators between the microfluidic chip and the enzyme-linked immunosorbent assay (ELISA, the gold standard). The relevant results are presented in Figs. 6–9 and Table 6.

The results showed that for all detected antibodies (AgB IgG, Ag5 IgG, Em2 IgG, Em18 IgG, LPS IgM, LPS IgG, Omp25 IgM, and Omp25 IgG), both the positive and negative coincidence rates of the two methods exceeded 90%. Among these antibodies, the positive coincidence rates of Em18 IgG and LPS IgG both reached 100%, and the negative coincidence rate of Em18 IgG also achieved the optimal detection performance of 100%. The Kappa values of all antibody indicators ranged from 0.88 to 0.98, all significantly higher than the critical value of 0.75 for good consistency. Notably, Em18 IgG exhibited the highest Kappa value of 0.98, indicating an almost perfect consistency between the two detection methods for this indicator.

The Bland-Altman analysis demonstrated that the proportion of samples falling within the 95% confidence interval for each indicator was no less than 90%, among which, LPS IgG showed the highest proportion of 95%. Although the proportions for Omp25 IgM and Omp25 IgG were 90%, the lowest among all indicators, they still met the clinically acceptable criteria for the consistency evaluation. Linear regression analysis revealed that the coefficients of determination (R^2^) for all eight indicators were greater than 0.99. Combined with the scatter plots and the aforementioned consistency evaluation indicators, it can be concluded that the detection results of the microfluidic chip and the ELISA method have excellent consistency, and the detection differences between the two methods are within the clinically acceptable range. These results further confirm the reliability and validity of the microfluidic chip in the detection of the above-mentioned antibodies.

## Discussion

In this study, a magneto-immunofluorescent microfluidic chip was successfully developed and fabricated, enabling the simultaneous and rapid detection of serum antibodies against brucellosis and echinococcosis. A systematic and comprehensive verification of all core detection performances of the chip was conducted, and a consistency comparison analysis was performed between its detection results and those of the enzyme-linked immunosorbent assay (ELISA), the clinical gold standard. Experimental results demonstrated that the chip could achieve stable and reliable detection of eight target antibodies, namely AgB IgG, Ag5 IgG, Em2 IgG, Em18 IgG, LPS IgM, LPS IgG, OMP25 IgM, and OPM25 IgG, with the chip’s key performance indicators including linear range, limit of detection, precision, and specificity all meeting the relevant criteria for clinical detection. Furthermore, the chip exhibited a high degree of consistency with the ELISA method in both qualitative and quantitative detection. This finding fully confirms that the proposed technology can serve as an alternative or supplementary approach to traditional detection methods for the dual screening of brucellosis and echinococcosis. The findings of this study also provide a novel technical method for the simultaneous detection of zoonotic co-infections in pastoral areas with inadequate medical resources.

The high consistency of detection results between the developed chip and the ELISA method serves as the core evidence for the clinical application potential of the chip, as the positive and negative coincidence rates of all target antibodies in this study exceeded 90%, with the positive coincidence rates of Em18 IgG and LPS IgG reaching 100% and the negative coincidence rate of Em18 IgG also attaining 100%; the Kappa values of all indicators ranged from 0.88 to 0.98, far higher than the critical value of 0.75 for good consistency, and more than 90% of sample results fell within the 95% confidence interval in the Bland-Altman analysis. Notably, the outstanding clinical detection performance of the chip is fundamentally determined by the high coupling efficiency between antibodies/antigens and microspheres. After systematic optimization of mass conjugation ratios from 10:1 to 100:1, the optimal mass ratio of antibody to fluorescent microspheres was identified as 25:1, and that of antigen to magnetic beads as 50:1, with corresponding coupling efficiencies of 89.4% and 89.1%, respectively (*i.e.*, over 89% of antibodies or antigens can be successfully immobilized on microsphere surfaces). This critical parameter directly dictates the stability and superior analytical performance of the detection system. Specifically, the high coupling efficiency of 89.4% ensures abundant effective antibody loading on fluorescent microspheres, eliminating inadequate capture antibodies caused by incomplete conjugation, substantially boosting the capture efficiency of low-abundance target antibodies in serum, reducing the limit of detection (LOD), facilitating early clinical screening and minimizing missed diagnoses—an advantage vital for the early screening of acute brucellosis and mild echinococcosis among pastoral populations. Meanwhile, the 89.1% coupling efficiency between antigens and magnetic beads enhances the specific binding affinity of magnetic beads to target antibodies and alleviates non-specific adsorption competition triggered by insufficient immobilized antigen, raising detection specificity to over 98% and cutting down clinical false-positive rates. In pastoral areas with a high incidence of co-infections, this merit prevents patients from receiving redundant treatment due to misdiagnosis and eases the workload of primary medical institutions. This excellent consistency originates from the synergistic effect of the chip’s integrated technical system: magnetic immunoisolation technology efficiently enriches trace target antibodies in serum and reduces interference from heteroproteins, laying a solid foundation for the accuracy of detection results (Kang et al., 2024); the micron-scale integrated channels of the microfluidic chip standardize the antigen-antibody binding and fluorescent detection reactions, preventing the human operational errors often seen in the traditional ELISA method and ensuring the repeatability of detection (Cao et al., 2023; Thomas et al., 2023); and the high sensitivity of immunofluorescence detection further improves the quantitative accuracy of the chip, resulting in a high degree of agreement between its results and the results of the gold standard (Sheng et al., 2023; Skourti-Stathaki, 2022). Compared with existing single-target microfluidic detection technologies for brucellosis or echinococcosis, the chip developed in this study achieves simultaneous detection of the two zoonotic diseases while maintaining high detection performance, effectively addressing the low screening efficiency caused by separate detection in areas with a high incidence of co-infections, making the chip more compatible with the practical demands of epidemiological surveys and large-scale population screening. In addition, the magneto-immunofluorescent microfluidic chip exhibits distinct technical advantages over the traditional ELISA method. These advantages are particularly prominent in the practical scenarios of zoonotic disease prevention and control. First, its “one-chip multi-test” mode breaks the limitation of single-pathogen detection, enabling the simultaneous detection of multiple antibodies of the two diseases with a single sample, greatly improving detection efficiency by being suitable for the rapid screening of suspected and high-risk populations in pastoral areas. Second, the chip integrates sample processing, immune reaction, and signal detection, and can be operated with simple equipment such as a centrifuge, simplifying the professional operational requirements of the traditional ELISA method and making it feasible for primary medical institutions and on-site testing points in pastoral areas to conduct relevant detection work. Third, the chip requires an extremely small serum sample volume for detection, alleviating the burden of sample collection on patients and being more applicable to special groups such as children and the frail in pastoral areas (Jeon et al., 2025; Matson, 2023; Postek, Pacocha & Garstecki, 2022; Wu et al., 2023). Furthermore, the detection time of the chip is significantly shorter than that of the traditional ELISA method, which would help improve the early intervention of patients with brucellosis and echinococcosis, the prevention of chronic progression of acute brucellosis, and early treatment for echinococcosis patients with mild cyst lesions. An important innovation of this study lies in the integration of magnetic immunoisolation, immunofluorescence detection, and microfluidic chip technologies. This technical combination also provides a valuable reference for the development of multiplex detection technologies for other zoonotic diseases. At present, most microfluidic detection technologies for zoonotic diseases adopt a single immunoassay mode, which suffers from insufficient detection sensitivity for trace antibodies in serum samples due to the lack of a target enrichment step; in this study, the combination of magnetic immunoisolation and immunofluorescence detection realizes the organic integration of target enrichment and high-sensitivity quantitative detection, effectively improving the chip’s detection performance for low-concentration antibodies. This technical strategy can be extended to the detection of other zoonotic diseases with insidious early symptoms and low antibody concentrations in the early stage of infection, thus providing a new research idea for the construction of a simultaneous multi-pathogen screening platform based on microfluidic chip technology.

**Table 5:** Mean values and Relative Standard Deviations (RSD) of antibody levels in two samples.

**Sample number**	**AgB IgG**		**Ag5 IgG**		**Em2 IgG**		**Em18 IgG**		**LPS IgM**		**LPS IgG**		**Omp25 IgM**		**Omp25 IgG**	
	**Mean value**	**RSD (%)**	**Mean value**	**RSD (%)**	**Mean value**	**RSD (%)**	**Mean value**	**RSD (%)**	**Mean value**	**RSD (%)**	**Mean value**	**RSD (%)**	**Mean value**	**RSD (%)**	**Mean value**	**RSD (%)**
1	11.8	4.01	15.93	3.77	7.99	2.85	18.08	2.78	5.99	4.83	9.05	4.51	9.87	5.11	11.18	3.14
2	59.29	2.82	71.88	3.07	47.79	2.88	84.48	3.18	66.14	3.98	73.74	3.16	84.89	5.77	57.97	4.07

**Table 6:** Coincidence rates, kappa values, and Bland-Altman 95% CI sample proportions for IgG and IgM antibody detection assays.

**Item**	**Coincident rate positive**	**Coincident rate negative**	**Kappa**	**Proportion of samples within 95% CI of Bland–Altman analysis (%)**
AgB IgG	96% (48/50)	92% (46/50)	0.88	91% (91/100)
Ag5 IgG	98% (49/50)	96% (48/50)	0.94	93% (93/100)
Em2 IgG	96% (48/50)	98% (49/50)	0.94	93% (93/100)
Em18 IgG	98% (49/50)	100% (50/50)	0.98	94% (94/100)
LPS IgM	94% (47/50)	98% (49/50)	0.92	91% (91/100)
LPS IgG	100% (50/50)	95% (47/50)	0.94	95% (95/100)
OMP25 IgM	96% (48/50)	94% (47/50)	0.90	90% (90/100)
OMP25 IgG	92% (46/50)	96% (48/50)	0.88	90% (90/100)

This study has several limitations, which also provide specific directions for further optimization and in-depth research. First, the performance verification of the chip was only conducted with a limited number of serum samples, and the study subjects did not include individuals from different epidemic regions, age groups, and disease stages. Thus, the universality of the chip’s detection performance still requires multi-dimensional validation with a larger sample size. Second, the temperature difference fluctuates greatly in field detection scenarios of pastoral areas, and the stability of the chip’s detection precision in such environments has not been fully verified, so its environmental adaptability for practical applications in pastoral areas needs to be further evaluated. Third, the preparation process of the chip is still at the laboratory research stage, and the production cost is not optimal. Further reducing the manufacturing cost is key to realizing the large-scale promotion of this technology in primary medical institutions in pastoral areas.

In response to the above limitations, future research will focus on three core aspects: improving universality, optimizing environmental adaptability, and controlling industrial production costs. First, the collection of serum samples will be expanded and a multi-center clinical verification will be carried out in different pastoral areas of China, including northern Xinjiang, Inner Mongolia, and Xizang. Individuals of different ages, disease stages, and co-infected populations will be enrolled to comprehensively validate the detection efficiency of the chip in various practical application scenarios and further enhance its clinical applicability. Second, verification tests on the chip’s detection precision will be performed in special environments with large temperature differences in pastoral areas. By optimizing the substrate material and immune reaction system of the chip, the environmental tolerance will be strengthened to ensure stable detection performance during field detection in pastoral areas. Third, the chip preparation process will be improved, and large-scale manufacturing approaches, such as injection molding and batch micro-nano processing, will be explored. The production cost can be effectively reduced through process upgrading, laying a solid cost foundation for the popularization and application of the chip in primary medical institutions of pastoral areas. In addition, the scope of detection targets will be further expanded based on the existing chip platform, indicators of other common zoonotic diseases in pastoral areas will be incorporated, a multi-target integrated rapid screening platform will be constructed, and the comprehensive prevention and control technical support capability of the chip will be strengthened for zoonotic diseases in pastoral areas.

## Conclusions

In summary, the magneto-immunofluorescent microfluidic chip developed in this study exhibits excellent brucellosis and echinococcosis detection performance with results highly consistent with the detection results of the clinical gold standard ELISA method, enabling the rapid and simultaneous detection of serum antibodies against these zoonotic diseases. This technology effectively overcomes the limitations of traditional detection methods, such as cumbersome operation and single-disease detection, and demonstrates significant application value in the on-site rapid screening and epidemiological surveillance of brucellosis and echinococcosis in pastoral areas. After further optimization and improvement, the chip is expected to provide critical technical support for the early diagnosis and precise prevention and control of brucellosis and echinococcosis in pastoral areas of China and other high-prevalence regions worldwide, thereby improving public health and preventing economic losses in the animal husbandry industry in regions with high-prevalence of these two diseases.

##  Supplemental Information

10.7717/peerj.21509/supp-1 Supplemental Information 1 Raw data

10.7717/peerj.21509/supp-2 Supplemental Information 2 Codebook

## Additional Information and Declarations

### Data Availability

The following information was supplied regarding data availability: The raw measurements are available in the Supplemental Files.
