# Supplementary material for: Magnetic immunofluorescent microfluidic chips for rapid multi-parameter detection of serum antibodies to brucellosis and echinococcosis
Source: PeerJ. 2026 Jul 3;14:e21509. doi: 10.7717/peerj.21509 (PMC13335499; doi:10.7717/peerj.21509)
Supplement: Supplemental Information 2 [file peerj-14-21509-s002.docx]

1. Manual Overview

This codebook focuses on the multiple verification methods adopted in the study (including ROC curve analysis, dose-response curve validation analysis, consistency analysis, and precision analysis). It clarifies the coding implications of the detection results for each method, so as to ensure the authenticity and reproducibility of the data.

1. Coding Meaning Table

2.1 Experimental Grouping Coding for ROC Curve Analysis

| Coding | Meaning |
| --- | --- |
| Sample 1~100 | Serum detection results of 100 individuals |
| ELISA | Detection results using the ELISA method |
| Chip | Detection results using the microfluidic chip method |

| 2.2. Grouping Coding for Consistency Analysis ELISA Results |
| --- |

| Coding | Meaning |
| --- | --- |
| Sample 1~50 | Serum detection results of 50 individuals |
| ELISA | Detection results using the ELISA method |
| Chip | Detection results using the microfluidic chip method |

| 2.3. Grouping Coding for Dose-Response Curve Detection Results |
| --- |

| Coding | Meaning |
| --- | --- |
| ELISA | Detection results of ELISA standards |
| Chip | Fluorescence intensity results of ELISA standards detected by microfluidic chip |

| 2.4. Grouping Coding for Precision Test Results |
| --- |

| Low-value sample | Detection results of low-value samples |
| --- | --- |
| High-value sample | Detection results of high-value samples |
